# Construction Products between Testing Laboratory and Market Surveillance: Case study of Cementitious Ceramic Tile Adhesives

**DOI:** 10.3390/ma15176167

**Published:** 2022-09-05

**Authors:** Cristina Stancu, Dawid Dębski, Jacek Michalak

**Affiliations:** 1 Ceprocim S.A., 6, Blvd. Preciziei, Sector 6, 062203 Bucharest, Romania; 2 Research and Development Center, Atlas sp. z o.o., 2, Kilińskiego St., 91-421 Lodz, Poland; 3 Izohan sp. z o.o., 2, Łużycka St., 81-963 Gdynia, Poland

**Keywords:** ceramic tile adhesive (CTA), assessment and verification of constancy of performance (AVCP), construction product, market surveillance, interlaboratory comparison (ILC), proficiency testing (PT), risk analysis, measurements uncertainty (MU)

## Abstract

This article presents the results of the interlaboratory comparison (ILC) study of the following four characteristics of ceramic tile adhesives (CTAs): initial tensile adhesion strength, tensile adhesion strength after heat ageing, tensile adhesion strength after immersion in water, and tensile adhesion strength after freeze–thaw cycles. The results showed that the objective of the ILC was achieved—the z-score analysis carried out following ISO 13528 allowed for classifying all results obtained by 23 laboratories out of 27 as satisfactory. The results of the remaining four laboratories were rated worse. Despite the achieved goal, the ILC notes high heterogeneity of the results in terms of failure patterns, as well as significant differences between the lowest and the highest values of tensile adhesion strength for various measurement conditions. The results of the ILC were discussed in terms of the possibility of including them in the risk analysis conducted by the manufacturer. The results of the ILC are also valuable information for market surveillance authorities, who, in the authors’ opinion, should be more cautious about results on samples taken from the market. The ILC results for CTAs are also a valuable recommendation for a possible revision of EN 12004.

## 1. Introduction

The rules for placing construction products on the market or making them available on the EU market are defined from 1 July 2013 in the Construction Products Regulations [1]. A construction product covered by a European harmonized standard [2] or compliant with an issued European Technical Assessment (ETA) [3] may be placed on the market or made available on the need of a given EU member state after its manufacturer has carried out its assessment and verification of constancy of performance (AVCP). As a result of the AVCP, the CE marking is applied.

As a rule, the basis for standardization work is transparency and openness of its conduct, aimed at achieving consensus, consistency, and independence from special interests effectively [4]. In principle, in the EU, regardless of the standardization organization, they are carried out by, i.e., CEN (Comité Européen de Normalization), CENELEC (Comité Européen de Normalisation Electrotechnique), and EOTA (European Organisation for Technical Assessment); the standards are driven by business with the involvement of various stakeholders in the stimulation of competition and innovation as improvement in consumer safety [5]. One of the European systems of technical regulations tasks is making European standards a global benchmark [6]. 

In its assumptions, standardization works use proven achievements of science and technology. The final result of work, which is a standard, is developed under the conditions of consensus. Still, what is natural is that the standard is perceived differently by various stakeholders, i.e., standardization bodies, authorities—primarily market supervision science, customers, and industry [7]. It is also important to note that the implementation of standards in organizations often remains a delicate topic [8]. Standardization is both an engine of progress and a potential barrier to it. The expenses of the production’s standardization process that adapts innovation to production are significant, mainly when an unskilled workforce is used [9]. However, above all, standards play an essential role in the quality of life and economy in the contemporary globalizing world [8]. A standard should take the interrelationships between science and practice. The classic linear innovation model, combining applied research with basic research, experimental development, production, and diffusion, does not necessarily apply to construction products. It often corresponds, in fact, to three scientific communities with their concepts [10].

The AVCP of construction products is performed following one of the five systems in Annex V of the CPR. AVCP systems define the catalog of activities that the external bodies are obliged to perform, i.e., the notified product certification body (system 1+, 1, or 2+) and the notified testing laboratory (system 3) participating in the product assessment procedure [1]. The manufacturer participates in all systems [1]. In addition, when considering AVCP, one should remember the role of the legislator who assigned specific tasks to market surveillance authorities, including construction supervision authorities [11] and science as the type of human knowledge that most adequately describes reality.

As mentioned above, the testing laboratories have their own goals and priorities, which could not be of primary importance to the product manufacturer or market surveillance authority. Proficiency Testing (PT)/Interlaboratory Comparison (ILC) is one of the essential instruments for quality control and assessment of competencies for testing laboratories [12,13,14]. Participation in PT/ILC allows a laboratory to compare its test results with the results of other laboratories, eliminate errors, verify staff competencies, and perform corrective actions. Participation in PT/ILC and activities undertaken by testing laboratories creates opportunities for increasing customer confidence in the laboratory through the independent confirmation of the laboratory’s competence. Participation in PT/ILC should be regular, and it improves the functioning of laboratories [15,16]. In situations where PT/ILC are not available, alternative solutions are sought [17]. The general principle of PT/ILC is simple: the organizer prepares and provides participants with validated test material and the necessary information to perform the tests, collects the obtained test results, prepares the report, and communicates it to the participants. An important aspect of PT/ILC is respect for confidentiality by assigning code numbers to participants. Each laboratory knows its results, the list of participants, and the report but cannot identify the results of other participants.

The proper conduct of the PT/ILC and drawing the right conclusions in certain situations is difficult and requires special attention from the organizer. This is the case, among other things, if the participant number of PT/ILC is small [18,19]. Measurements of construction products and, thus, carrying out PT/ILC, due to the multi-stage nature of the test procedures, product heterogeneity, and when the methods used are destructive tests, are additionally complex. Thus, the conformity assessment is even more difficult—it often raises the question of to what extent the evaluation of the construction product based on the tests is reliable in the case of construction products [20,21,22]. Notably, the opinion of the laboratory often plays a decisive role—this is the case in the assessment of construction products collected from the market by market surveillance authorities and checking whether the product meets the parameters declared by the manufacturer [19,23,24]. 

All measurements are inextricably linked to reproducibility, uncertainty, and confidence issues [25]. The vast majority of measurements are performed for compliance assessment [26,27]. Less is written in the scientific literature about other uses of PT/ILC results, such as support for R&D, manufacturing, and operations [16,28,29]. In the traditional uncertainty approach, there is an inherent part of deviation of the observed characteristic. The definition of measurement uncertainty (MU), referring to the condition of being uncertain, is still the subject of many considerations by metrology specialists [25,30]. One of them is the statement that MU is a descriptive parameter of what one knows that one does not know [30]. 

The problem of MU is complex [31]. Thus, the large MU often makes the acceptance zone small or very small, and sometimes also completely eliminated [32]. The fact that the MU is small does not mean that the laboratory performing the measurement is better than another. Due to the multi-stage nature of the test procedures, and the construction product’s heterogeneity, most construction product tests result in a low MU value from not taking into account all the components influencing the uncertainty [33]. Regardless of various observations and doubts, the MU paradigm forms the basis for assessing compliance. It is necessary to explain the influence of all sources of systematic and random effects on the measurement [34]. The probability of making a wrong decision depends on the size of the MU and how the uncertainty is considered when assessing compliance [26,35]. Due to the variance in the measured characteristics of the product, there is always a risk of incorrect assessment. A product assessed as compliant may be non-compliant, and a product rejected as non-compliant may actually be a compliant product [35,36].

From the producer’s perspective, the reproducibility of the result is crucial when the product is reassessed by market surveillance authorities, i.e., the exact product tested with the same method in different laboratories, by other operators, and with various equipment. It becomes vital when the actual values of the product’s performance properties are close to the standard limit values.

When considering the issues of PT/ILC, MU, and their impact on conformity assessment, one should always bear in mind the economic dimension related to the statement that a given product is compliant or non-compliant [29,37,38,39].

This article presents the results of the 12th edition of the ILC for cementitious ceramic tile adhesives (cementitious CTAs) conducted in 2020–2021 by Ceprocim (EU-notified laboratory no. 1830). The ILC for cementitious CTAs was first organized by Ceprocim in 2008 [40] and is today a recognized and respected study in the European CTA community. It should be added that in the field of construction products, there is a minimal offering in the area of PT/ILC (study of the internet resources by keywords: “interlaboratory comparisons” or “proficiency testing” reveals many possibilities for performing PT/ILC in microbiology, food or clinical research). According to the assumptions of the organizers of the discussed ILC, it was aimed at evaluating the competencies of the participating laboratories, helping to identify existing problems, and educating the staff. In the case of accredited laboratories, the ILC is an additional opportunity to confirm that activities following the requirements of EN ISO/IEC 17,025 are conducted [41].

Ceramic tiles are widely used all over the world. In 2020, 16.093 billion m^2^ was produced, while slightly less was used—16.035 billion m^2^ [42]. Assuming all ceramic tiles are installed using CTAs, this means a consumption of around 65 million tons of CTAs [43]. Asia produced the most significant quantity of ceramic tiles—11.905 billion m^2^ (74.0% of global production), of which 8.474 billion m^2^ was in China, and Asia consumed the most—11.470 billion m^2^. In the EU countries, ceramic-tile production was equal to 1.218 billion m^2^, and 1.035 billion m^2^ was installed [42].

Before 2001, there were no detailed requirements applicable to all construction market participants in the EU countries in introducing CTAs. It made it difficult for both the investor and the contractor to choose the right CTA and made it difficult, and often impossible, to evaluate the product objectively in comparison to other CTAs offered. In 2001, EN 12004:2001, developed in CEN/TC 67/WG 3, was established [44]. This standard has been amended several times over the past twenty years. Its latest version is EN 12004-1:2017 [45], but the basis for AVCP is still EN 12004:2007+A1:2012 [46]. The reason is that the 2017 version has not been published in the list of European harmonized standards in the *Official Journal of the European Union* [2].

The EN 12004 standard, apart from the requirements for cementitious CTAs, also specifies requirements for dispersion and reaction resin CTAs. Following the requirements of EN 12004, cementitious CTAs are divided into two classes: C1, for which values of initial tensile adhesion strength, tensile adhesion strength after water immersion, tensile adhesion strength after heat ageing, and tensile adhesion strength after freeze–thaw cycles are 0.5 N/mm^2^; and C2, for which the values of all the aforementioned characteristics are 1.0 N/mm^2^.

The International Organization for Standardization (ISO), a standardization organization with a much larger scope than CEN (currently associated with 162 national standardization organizations), adopted the assumptions of EN 12004. ISO in 2004 established the ISO 13007-1 standard [47], and, thus, the requirements proposed by CEN/TC 67/WG 3 have become widespread worldwide. The current version of ISO 13007-1 comes from 2014 [48].

The results obtained in the ILC were discussed traditionally, i.e., with the use of statistical methods intended for this type of research. Additionally, the obtained results were related to the assessment made by the construction supervision authority when taking construction products from the market and performing their reassessment. Such a non-stereotypical approach helped formulate recommendations regarding the desired changes in the standard for CTAs.

## 2. Materials and Methods

Ceprocim, in 2020–2021, organized the ILC of CTA tests for the twelfth time. Twenty-seven laboratories from the following countries participated in the 12th edition: Austria (2), France (1), Germany (3), Greece (2), Hungary (1), Italy (3), Mauritius (1), Netherlands (1), Poland (1), Portugal (1), Republic of Moldova (1), Romania (6), Slovenia (1), Spain (2), and United Arab Emirates (1). Most of them were accredited laboratories according to EN ISO/IEC 17025. Most of the 27 laboratories participating in the ILC were research and development institutes dealing with measurements, including laboratories notified in the scope of EN 12004. The remaining laboratories belonged to producers of CTAs (large companies with an international range of activity) or producers of chemical additives for modification of CTAs (also significant global concerns acting around the world). Most of the 27 laboratories participated in previous editions of the ILC organized by Ceprocim.

In ILC, defining the property or properties to assess with the homogeneity check is essential. For the ILC described in this article, the residue test on the 250 μm sieves was performed to establish CTA homogeneity. ILC organizer made the tests on different samples from the CTA subject to homogenization. Testing the CTA homogeneity was performed with the same equipment, by the same operator, in a short period. The sample was considered homogeneous when all the results had been placed in the range: average value of the residues on the 250 μm sieve ± 2s (%), where s represents the standard deviation of repeatability. The value of s represents the standard deviation of repeatability.

The ILC organizer provided each participating laboratory with the CTA samples (CTA class C2) for testing, ceramic tiles (according to the test method specified in Table 1 of EN 12004:2007+A1:2012) and all the necessary instructions to complete the task. All determinations, i.e., the initial tensile adhesion strength, tensile adhesion strength after water immersion, tensile adhesion strength after heat ageing, and tensile adhesion strength after freeze–thaw cycles, were made following the requirements of EN 12004:2007+A1:2012 and the standards describing the test methods referred to in this standard [46]. In short, the procedure for preparing CTA samples for testing by each laboratory was to apply a layer of the CTA to the concrete slab and then place test tiles. After preparation, test samples were stored under certain conditions, and then the pull-head plates were bonded to the tiles with epoxide adhesive (higher-strength adhesive than tested CTA). After additional storage under specified conditions, the tensile adhesion strength of the CTA by applying a force increasing at a constant rate of (250 ± 50) N/s was measured.

Apart from the CTA mentioned above, the test sample and ceramic tiles were provided by the ILC organizer; all other auxiliary materials necessary for these tests, such as concrete slabs, water, and measuring instruments, were provided by individual laboratories.

The ILC organizer performed statistical calculation according to the ISO 13528:2015 [49] with algorithm A described in Annex C (clause C.3). It implies, for initial adhesion strength, tensile adhesion strength after heat ageing, tensile adhesion strength after water immersion, and tensile adhesion strength after the freeze–thaw cycle, a calculation of the robust values for average and for standard deviation from the results obtained of each participant. 

An iterative calculation derived the robust average (x*) and the robust standard deviation (s*), i.e., by updating the values of x* and s* several times using the modified data until the process converges. Convergence was assumed when there was no change from one iteration to the next in the third significant figure of the robust mean and standard deviation (x* and s*). The value obtained for the robust average after the last iteration represents the assigned value (*x_pt_*), chosen to be the consensus value. 

The standard uncertainty *u*(*x_pt_*) of the assigned value was calculated following the formula presented in Equation (1):

*u*(*x_pt_*) = 1.25 × *σ_pt_/*√*p* (1)

where: 

*σ_pt_*—standard deviation for proficiency assessment;

*p*—the number of participant laboratories that carried on the test on a concrete slab. 

The z-score was calculated with the formula given in Equation (2):

*_Zi_* = *x_i_* − *x_pt_*/*σ_pt_* (2)

where: 

*x_i_*—the value obtained by each participant for each test; 

*x_pt_*—the assigned value on total participants for each test.

The obtained results were evaluated according to EN ISO/IEC 17043:2010, clause B.4. Following B.4.1.1. of EN ISO/IEC 17043:2010 for the z indicator, the commonly used assessment was applied, i.e.,

- ǀzǀ ≤ 2—satisfactory; therefore, it does not trigger any warning signal or signal for action;
- 2 < ǀzǀ < 3—questionable, it causes a warning signal;
- ǀzǀ ≥ 3—unsatisfactory, triggers an action signal.

In the z-score calculation program, the assigned value and the robust standard deviation value obtained after the last iteration were used as they result from calculation without being round. 

## 3. Results

The results of initial tensile adhesion strength and tensile adhesion strength after heat ageing of CTA are presented in Table 1. Table 2 summarizes the results obtained for CTA measurements of tensile adhesion strength after water immersion and tensile adhesion strength after freeze–thaw cycles. Table 1 and Table 2 also list the dominant failure pattern observed for each measurement of tensile adhesion strength. Possible failure patterns are described in clause 3.6 of EN 12004:2007+A1: 2012 and presented graphically in Annex 1 to this standard [46].

Table 3 summarizes the lowest and the highest values obtained for all measured characteristics in the ILC.

Table 4 presents a summary of the predominant mode of failure obtained for each of the measured characteristics.

**Table 1:** The initial tensile adhesion strength and tensile adhesion strength after heat ageing of CTA with the predominant mode of failure obtained by 27 laboratories (participant codes from 1 to 27) in the 12th ed. of ILC.

Participant Code	Initial Tensile Adhesion Strength		Tensile Adhesion Strength after Heat Ageing	
	[N/mm^2^]	Dominant Failure Pattern	[N/mm^2^]	Dominant Failure Pattern
1	1.5	CF-A	1.5	AF-T
2	1.4	50% CF-A/50%AF-T	1.3	50% CF-A/50% AF-T
3	1.6	CF-A	1.6	CF-A
4	1.7	CF-A	1.8	CF-A
5	1.9	CF-A	1.3	CF-A
6	2.0	CF-A	1.5	CF-A
7	1.9	CF-A	2.0	CF-A
8	1.3	CF-A	1.6	CF-A
9	1.6	CF-A	1.3	CF-A
10	1.6	CF-A	1.5	CF-A
11	1.3	AF-S	0.6	CF-A
12	1.5	AF-T	1.3	AF-T
13	1.8	CF-A	1.9	CF-S
14	2.4	AF-S	2.1	AF-T
15	1.9	CF-A	1.8	CF-A
16	1.9	50% CF-A/50% AF-T	1.1	55% CF-A/45% AF-T
17	2.7	CF-A	2.5	CF-A
18	1.6	AF-S	0.8	BT
19	2.0	CF-A	1.7	CF-A/AF-S
20	1.5	AF-T	2.3	AF-T
21	2.0	CF-A	1.9	CF-A
22	2.0	CF-A	1.8	CF-A
23	1.4	AF-T/CF-A	1.7	CF-A
24	1.5	CF-A	1.8	CF-A
25	2.3	AF-T	1.6	AF-T
26	2.3	CF-A	2.1	CF-S
27	1.7	CF-A	2.2	CF-A

CF-A—cohesive failure within the adhesive, AF-T—adhesion failure between adhesive and tile, AF-S—adhesion failure between adhesive and substrate, CF-S—cohesive failure in the substrate, CF-T—cohesive failure in the tile or BT—adhesive failure between tile and pull head plate.

**Table 2:** The tensile adhesion strength after immersion in water and tensile adhesion strength after freeze–thaw cycles of CTA with the predominant mode of failure obtained by 27 laboratories in the 12th ed. of ILC.

Participant Code	Tensile Adhesion Strength after Immersion in Water		Tensile Adhesion Strength after Freeze–Thaw Cycles	
	[N/mm^2^]	Dominant Failure Pattern	[N/mm^2^]	Dominant Failure Pattern
1	0.9	AF-T	1.5	AF-T
2	0.5	5% CF-A/95%AF-T	0.8	50% CF-A/50% AF-T
3	0.8	CF-A	1.0	CF-A
4	1.0	CF-A	1.3	CF-A
5	0.6	AF-T	0.9	CF-A
6	1.0	AF-T	1.2	AF-T
7	0.6	CF-A	1.4	CF-A
8	1.1	CF-A	1.6	CF-A
9	1.1	CF-A	1.2	CF-A
10	0.9	AF-T	1.0	AF-T
11	0.6	AF-T	0.1	AF-T
12	0.4	AF-T	*	*
13	0.9	CF-A	1.2	CF-A
14	1.3	AF-T	0.6	CF-S
15	1.5	CF-A	1.6	CF-A
16	0.6	20% CF-A/80% AF-T	1.2	65% CF-A/35% AF-T
17	1.1	CF-A	2.1	CF-A
18	1.2	CF-A	0.3	AF-T
19	1.5	CF-A/AF-S	2.1	CF-A
20	1.2	AF-T	1.1	AF-T
21	1.1	CF-A	1.6	CF-A
22	0.6	CF-A	0.8	CF-A
23	0.6	AF-T	1.4	CF-A
24	0.8	AF-T	1.7	CF-A
25	0.9	AF-T	1.4	CF-A
26	1.1	AF-T	1.6	CF-A
27	2.0	CF-A	*	*

CF-A—cohesive failure within the adhesive, AF-T—adhesion failure between adhesive and tile, AF-S—adhesion failure between adhesive and substrate, CF-S—cohesive failure in the substrate, CF-T—cohesive failure in the tile or BT—adhesive failure between tile and pull head plate. * the laboratory did not report the results for this characteristic (25 laboratories reported results for the measurements of the tensile adhesion strength after freeze–thaw cycles).

**Table 3:** The lowest and highest values of the initial tensile adhesion strength, tensile adhesion strength after heat ageing, tensile adhesion strength after water immersion, and tensile adhesion strength after freeze–thaw cycles of CTA obtained by 27 laboratories participating in the ILC.

	Initial Adhesion Strength [N/mm^2^]	Adhesion Strength after Heat Ageing [N/mm^2^]	Adhesion Strength after Water Immersion [N/mm^2^]	Adhesion Strength after Freeze–Thaw Cycles [N/mm^2^]
Lowest value	1.3	0.6	0.4	0.1
Highest value	2.7	2.5	2.0	2.1

## 4. Discussion

The organizer of the ILC performed the statistical calculation of the results obtained by 27 participating laboratories according to the ISO 13528 [49]. Table 5 summarizes the results of the calculation.

The z-score values calculated following Equation (2) for each laboratory for the initial tensile adhesion strength, tensile adhesion strength after heat ageing, tensile adhesion strength after water immersion, and tensile adhesion strength after freeze–thaw cycles measurements are listed in Table 6.

The z-score analysis showed that among 27 laboratories participating in the ILC, 22 laboratories obtained satisfactory (ǀzǀ ≤ 2) results for all measured characteristics. One laboratory (code 12) obtained a satisfactory result for three measured characteristics (this lab did not provide a result for tensile adhesion strength after freeze–thaw cycles). Three laboratories marked as 11, 17, and 18 obtained a result classified as questionable (2 < ǀzǀ < 3) for the two measured characteristics. Only one laboratory (participant code 27) received a result that was considered unsatisfactory (ǀzǀ ≥ 3) for one measured characteristic (tensile adhesion strength after water immersion). The same laboratory did not provide the result for the characteristic of tensile adhesion strength after freeze–thaw cycles.

The results of the z-score analysis from the perspective of the laboratories participating in the ILC should be considered good, even very good. From this perspective, most laboratories fulfill expectations in their participation in the ILC. In addition, if we consider that among these 27 laboratories, 19 also participated in the previous edition of the ILC, and when comparing the results of only these 19 laboratories in two subsequent editions of the ILC, it can be concluded that in the following year, they obtained better results [16]. Comparing the results of these 19 laboratories with the entire sample of 27 laboratories also showed that the 19 obtained better results than the total 27. For the initial tensile adhesion strength measurements, all 27 laboratories obtained results from 1.3 N/mm^2^ to 2.7 N/mm^2^, while for 19 laboratories, this range was narrower and amounted to between 1.3 N/mm^2^ and 2.4 N/mm^2^. After immersion in water, the tensile adhesion strength measurement was 0.4–2.0 N/mm^2^ and 0.4–1.5 N/mm^2^, respectively. 

By analyzing the z-scores, we can indicate the leaders among the ILC participants. These are the laboratories labeled 1, 3, 4, 6, 10, and 13, and their results were the most consistent. However, the z-score analysis is one dimension of this study. When we look at the differences between the lowest and the highest measured values, they are significant. After all, 1.3 N/mm^2^ (the lowest value obtained in the case of the characteristic of initial tensile adhesion strength) is less than half of the importance of 2.7 N/mm^2^ (the highest value of the initial tensile adhesion strength). Even more significant differences were observed in the case of other characteristics, which is visible in Table 3. The analysis of the observed failure patterns (Table 4) also shows significant differences between the results obtained by individual laboratories. 

From the manufacturer’s perspective, placing a product on the market is associated with risks. Of course, this applies to a product such as the CTA. One of the risks is a negative assessment of the CTA in tests commissioned by market surveillance authorities. It is particularly probable when the actual values of the product’s performance properties are close to the standard assessment criterion’s limit value. For this reason, each responsible manufacturer carries out an uncertainty analysis. This analysis also considers the uncertainty associated with the measurements (measurement uncertainty). There is always a risk of incorrect assessment due to a variance in measured characteristics. The product assessed as compliant may be non-compliant, while the product rejected as non-compliant may actually be a compliant product. 

As in the case of many construction products, the determination of CTAs’ adhesion characterizes the multi-stage nature of the test procedures, affecting measurements and, of course, measurement uncertainty. There are test results available showing the influence of the concrete slab used for the tests [50] and the type of ceramic tile [51], and the type of water used to season the samples [52]. These studies showed that in some cases, the differences between the obtained test results are so significant that they could potentially be decisive in meeting the standard criteria [50,51,52]. 

**Figure 1:**
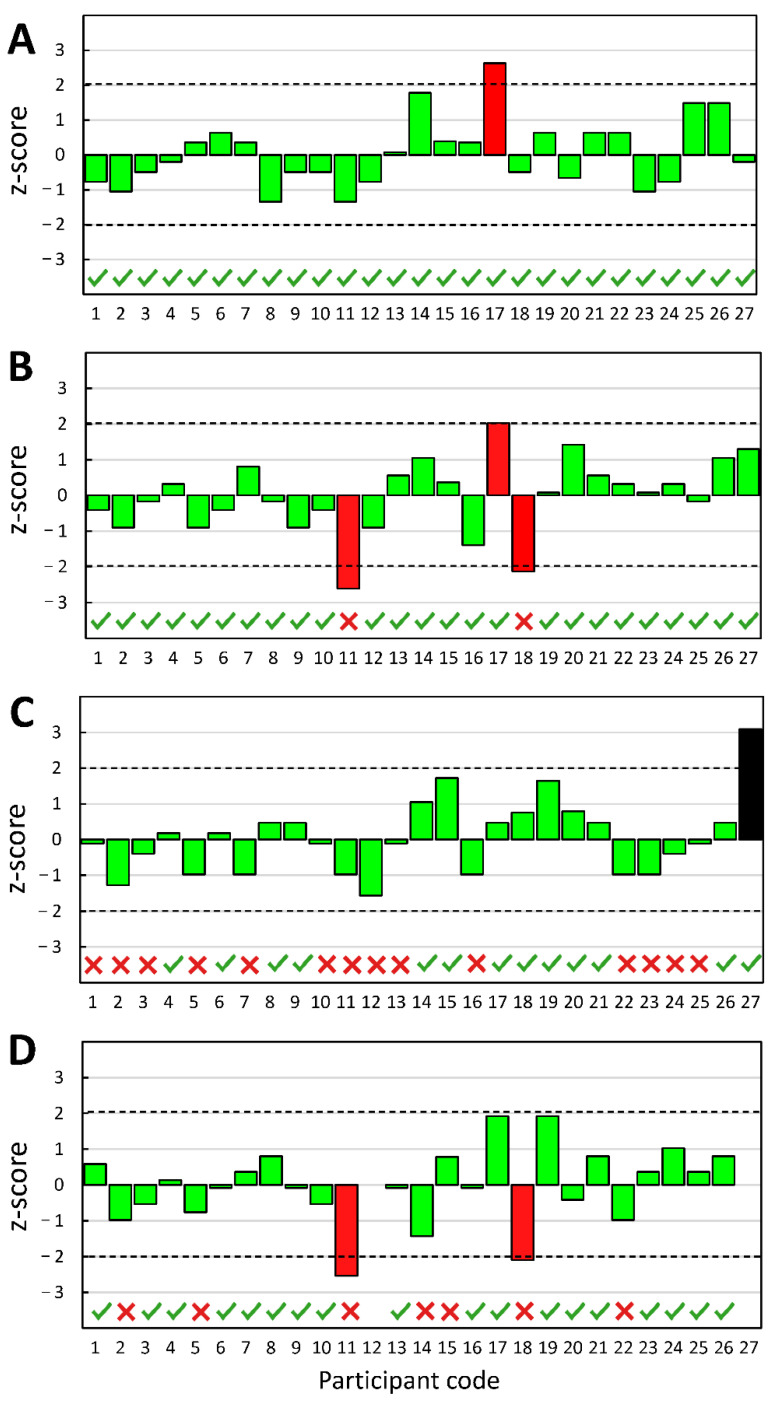
The results of the z-score analysis for measurements of the following CTA characteristics (**A**)—initial tensile adhesion strength, (**B**)—tensile adhesion strength after heat ageing, (**C**)—tensile adhesion strength after immersion in water, and (**D**)—tensile adhesion strength after freeze–thaw cycles. Legend: (■) |z| < 2 (satisfactory); (■) 2 < |z| < 3 (questionable); (■) |z| ≥ 3 (unsatisfactory); ( 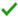) samples that were assessed by the construction supervision as meeting the requirements for CTA class C2 (>1.0 N/mm^2^ following EN 12004); ( 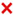) samples that the construction supervision has assessed as not meeting the requirements for CTA class C2.

The results of the ILC are valuable information for a manufacturer who carries out a risk analysis related to the introduction of a product. The results of the ILC are also a source of essential guidelines for possible amendments to standards. Additionally, market surveillance authorities can derive valuable information from the ILC results. Figure 1 shows the results of the z-score analysis for the characteristics tested during the ILC. Results of the z-score study are compared to the decisions made by market surveillance authorities during controlling CTAs from the market using the simple acceptance method that does not consider the variability resulting from MU.

The tensile adhesion strength test is widely used in construction, as is the pull-off technique. Variability of the pull-off method for adhesion strength evaluation is known [53]. Measurement of tensile adhesion strength represents a destructive test. Recently, Delgado et al. published a review on mortar bond tests [54]. The authors pointed out that the tensile adhesion strength test is characterized by a high variability in the obtained results also due to other inherent factors related to the procedure of application of CTAs and the equipment itself [54]. Delgado et al. suggested examining another characteristic parallel if the product is assessed using the tensile adhesion method [54]. The research results described in this paper are also entitled to such a postulate.

## 5. Conclusions

The analysis of the CTA tensile adhesion results performed by 27 laboratories participating in the ILC showed that the goal of the ILC/PT was achieved. Most laboratories (23) obtained results that, following the calculations made with ISO 13528 and EN ISO/IEC 17043 criteria [55], can be classified as satisfactory (z-score value: ǀzǀ ≤ 2). The results were questionable or not satisfactory in the case of 7 out of 106 measurements (z-score value: 2 < ǀzǀ < 3 (questionable) or ǀzǀ ≥ 3 (unsatisfactory). 

However, for all the tested CTA characteristics, i.e., initial tensile adhesion strength, tensile adhesion strength after heat ageing, tensile adhesion strength after immersion in water, and tensile adhesion strength after freeze–thaw cycles, the differences between the lowest and the highest values were significant (even the highest value was five times bigger than the lowest value). Similarly, the failure pattern analysis indicated a substantial heterogeneity of the results obtained.

The ILC for CTAs provides valuable information for the manufacturer who can use these results in a risk analysis. The ILC results show unequivocally that surveillance authorities should be more cautious about the consequences of CTA sample measurements taken from the market. The ILC results are also a recommendation for the authors of EN 12004 and standardization bodies to amend the requirements of this standard. In light of the results described in this article, it seems necessary to introduce EN 12004 rules specifying the need to include MUs in AVCP.

## Figures and Tables

**Table 4:** The number of the predominant mode of failure for the initial tensile adhesion strength, tensile adhesion strength after heat ageing, tensile adhesion strength after water immersion, and tensile adhesion strength after freeze–thaw cycles of CTA obtained by 27 laboratories participating in the ILC.

Predominant Failure Pattern	Initial Adhesion Strength	Adhesion Strength after Heat Ageing	Adhesion Strength after Water Immersion	Adhesion Strength after Freeze–Thaw Cycles
CF-A	18	16	12	16
AF-T	3	5	12	6
AF-S	3	0	0	0
CF-S	0	2	0	1
CF-T	0	0	0	0
BT	0	1	0	0
other	3	3	3	2

**Table 5:** The value of statistical parameters calculated following ISO 13528 for measurements of CTA initial tensile adhesion strength, tensile adhesion strength after heat ageing, tensile adhesion strength after water immersion, and tensile adhesion strength after freeze–thaw cycles.

Parameter	Initial Adhesion Strength [N/mm^2^]	Adhesion Strength after Heat Ageing [N/mm^2^]	Adhesion Strength after Water Immersion [N/mm^2^]	Adhesion Strength after Freeze–thaw Cycles * [N/mm^2^]
x*	1.7	1.7	0.9	1.2
s*	0.3	0.3	0.4	0.4
*x_pt_*	1.8	1.7	0.9	1.2
*_pt_*	0.4	0.4	0.3	0.4
*u*(*x_pt_*)	0.1	0.1	0.1	0.1
*V*	19.9	24.5	36.8	36.1

x*—robust average of the results reported by all participating laboratories; s*—robust standard deviation of the results reported by all laboratories; *x_pt_*—assigned value—consensus value; *σ_pt_*—standard deviation for proficiency assessment; *u*(*x_pt_*)—standard uncertainty of the assigned value; *V*—coefficient of variation. * 25 laboratories reported results for the measurements of the tensile adhesion strength after freeze–thaw cycles.

**Table 6:** The z-score values calculated for each participating laboratory for measurements of CTA initial tensile adhesion strength, tensile adhesion strength after heat ageing, tensile adhesion strength after water immersion, and tensile adhesion strength after freeze–thaw cycles.

Participant Code	Initial Adhesion Strength [N/mm^2^]	Adhesion Strength after Heat Ageing [N/mm^2^]	Adhesion Strength after Water Immersion [N/mm^2^]	Adhesion Strength after Freeze–thaw Cycles * [N/mm^2^]
1	−0.77	−0.41	−0.11	0.58
2	−1.05	−0.90	−1.27	−0.98
3	−0.49	−0.17	−0.40	−0.54
4	−0.20	0.32	0.18	0.13
5	0.36	−0.90	−0.98	−0.76
6	0.64	−0.41	0.18	−0.09
7	0.36	0.81	−0.98	0.36
8	−1.34	−0.17	0.47	0.80
9	−0.49	−0.90	0.47	−0.09
10	−0.49	−0.41	−0.11	−0.54
11	−1.34	−2.61	−0.98	−2.54
12	−0.77	−0.90	−1.56	-
13	0.08	0.56	−0.11	−0.09
14	1.78	1.05	1.05	−1.43
15	0.39	0.37	1.72	0.78
16	0.36	−1.39	−0.98	−0.09
17	2.63	2.03	0.47	1.92
18	−0.49	−2.13	0.76	−2.10
19	0.64	0.08	1.64	1.92
20	−0.66	1.42	0.79	−0.42
21	0.64	0.56	0.47	0.80
22	0.64	0.32	−0.98	−0.98
23	−1.05	0.08	−0.98	0.36
24	−0.77	0.32	−0.40	1.03
25	1.49	−0.17	−0.11	0.36
26	1.49	1.05	0.47	0.80
27	−0.20	1.30	3.09	-

* the laboratory did not report the results for this characteristic (25 laboratories reported results for the measurements of the tensile adhesion strength after freeze-thaw cycles).

## Data Availability

Not applicable.
